# CREATING DIGITAL STORIES ON AGING EXPERIENCES: A RAPID METHODOLOGICAL REVIEW OF DEVELOPMENT PROCEDURES

**DOI:** 10.1093/geroni/igae098.3832

**Published:** 2024-12-31

**Authors:** Nisha Gopalakrishnan, Katlyn Mellett, Noeman Mirza, Wendy Hulko, Daniel Gan, Karen Lok Yi Wong, Anthony L Kupferschmidt, Heather Lee

**Affiliations:** Thompson Rivers University, Kamloops, British Columbia, Canada; Thompson Rivers University, Kamloops, British Columbia, Canada; University of Winsdor, Toronto, Ontario, Canada; Thompson Rivers University, Kamloops, British Columbia, Canada; University of Toronto, Toronto, Ontario, Canada; University of British Columbia, Vancouver, British Columbia, Canada; City of Vancouver, Vancouver, British Columbia, Canada; City of Kamloops, Kamloops, British Columbia, Canada

## Abstract

Digital storytelling is increasingly used to showcase the life histories of older adults and their communities. The Mobilizing Aging in Place (MAP) project team was tasked to engage older adults in the creation of five digital stories as a means of sharing the findings of two studies on aging in place with municipal and regional planners and health care policy/decisionmakers. While there were numerous examples of digital stories on aging experiences, the best way to develop digital stories with older adults was unclear. To create clarity in effective methods, we conducted a rapid methodological review of existing literature on digital storytelling involving older adults and experiences of aging. After searching in the Discovery search engine, we reviewed 61 abstracts and articles based on specific inclusion and exclusion criteria. This yielded 18 articles that we included in the review. The included literature discussed digital stories on various health and intergenerational knowledge transfer topics. Researchers used a variety of software, editing tools, and recording methods to combine images, videos, and sounds. Digital stories were developed both in collaboration with older adults and by the older adult participants themselves. The review showed that there are no set criteria or best practices for creating digital stories, and that workshops and interviews are commonly used to engage older adults in digital storytelling. We recommend a more comprehensive and broader review be undertaken with a focus on all forms of digital storytelling regardless of type of population and context.

